# Benefits of autologous platelet tissue graft in wound healing after corneal refractive surgery: a case report

**DOI:** 10.1186/s13256-021-02694-9

**Published:** 2021-03-16

**Authors:** Yoshinori Takayanagi, Shigeaki Kato, Masaru Okada

**Affiliations:** 1 Takayanagi EYE Clinic, Sapporo S1 Building 3F, Nishi4-20-5, Minami1-jo, Chuo-ku, Sapporo, Hokkaido 060-0807 Japan; 2 Graduate School of Science and Engineering, Iryo Sosei University, 5-5-1, Iino, Chuo-dai, Iwaki, Fukushima 970-8551 Japan; 3 Research Institute of Innovative Medicine, Tokiwa Foundation, 57 Kaminodai, Jyoban Kamiyunagayamachi, Iwaki, Fukushima 972-8322 Japan

**Keywords:** Autologous platelet tissue graft, Gravitational platelet separation (GPS), Corneal wound healing, Photorefractive keratectomy (PRK), Laser *in situ* keratomileusis (LASIK)

## Abstract

**Background:**

Corneal refractive surgeries cause epithelial damage and induce wound healing processes. To promote wound healing after photorefractive keratectomy, the effectiveness of an autologous platelet tissue graft was assessed.

**Case presentation:**

A 45-year-old Asian male with low myopia and/or myopic astigmatism received photorefractive keratectomy in both eyes. The right eye was postoperatively treated with an autologous platelet tissue graft using the GPS III Platelet Concentration System to prepare platelet-rich plasma, while the left eye was not treated. Both eyes achieved the expected uncorrected distance visual acuity, but the platelet-rich-plasma-treated right eye obtained improved visual acuity more than 1 week before the untreated left eye. Perceived pain after photorefractive keratectomy was much lower and of shorter duration in the treated right eye than it was for the left eye. Pericorneal injection on the bulbar conjunctiva with superficial keratitis resolved earlier in the right eye than the left eye.

**Conclusions:**

Autologous platelet tissue grafting using a GPS III to obtain platelet-rich plasma was effective in promoting corneal wound healing after photorefractive keratectomy. Thus, platelet-rich plasma may be beneficial for patients undergoing corneal refractive surgeries.

## Background

Corneal refractive surgeries have been performed worldwide for patients with low myopia and/or myopic astigmatism to improve vision. Laser *in situ* keratomileusis (LASIK) has garnered widespread acceptance because of the minimal pain associated with the procedure and the prompt visual rehabilitation [1–3]. LASIK entails forming a hinged epithelial flap attached at the lower portion of the cornea using a laser or microkeratome, shaving the anterior corneal stroma, and replacing the flap over the corneal stroma. While LASIK is typically associated with a high level of safety and rapid healing owing to the absence of substantial stromal remodeling, the margin of the flap requires a localized but lengthy wound healing process for complete reorganization of the extracellular matrix layers [3–5], which can lead to flap-related complications. Due to inherent issues with the LASIK process, postoperative flap dislocation can also result from subsequent traumatic incidents.

Photorefractive keratectomy (PRK) emerged before LASIK, and this refractive surgery has been equally beneficial compared with LASIK for patients with low myopia and/or myopic astigmatism. PRK entails removing the cornea from the epithelial layers to the Bowman’s layer or the anterior stroma to the degree required for myopia correction [1–3]. The major difference between PRK and LASIK is thus the removal of surface cells from the cornea. Initially, a microkeratome was used for the removal process, but this has been replaced by lasers. More recently, the laser system has been improved by use of an excimer laser with a programmed ablation algorithm, resulting in reduced time and effort to perform the surgery and improved accuracy [1, 2]. Even with these improvements, the preservation of the corneal epithelium by PRK requires increased postoperative time for the wound healing process and increased pain compared with LASIK. Therefore, the promotion of wound healing would be a desirable benefit for PRK. The progress in understanding of the molecular mechanisms of inflammation and wound healing has led to development of postoperative treatments such as topical steroids and amniotic membrane patching [1–3]. As growth factors significantly facilitate wound healing processes [1–3], platelet-rich plasma (PRP) has been used in several applications for wound healing and tissue generation [6]. PRP is prepared from the plasma fraction of autologous blood of patients, avoiding any immune incompatibility [6].

The present study was thus conducted to determine if an autologous platelet tissue graft could promote wound healing after PRK, since autologous platelet tissue grafts have shown promise in the promotion of wound healing in many other surgical settings [6–9].

## Case presentation

A 45-year-old male received PRK using a MEL 90 excimer laser (Carl Zeiss Meditec, Jena, Germany) under the control of the triple-A algorithm[5] to correct myopia and myopic astigmatism in both eyes. Uncorrected distance visual acuity (UDVA) was 20/50 for each eye prior to the procedure. The astigmatism intensity was −0.75 for the right eye and −1.25 for the left eye, and a plano refraction was targeted under the auto-calculation system [5]. The PRK procedure started with a 20% alcohol treatment for 30 seconds using a standard ablation program with the optical zone set to 6.5 mm. After surgery, a bandage soft contact lens was attached and eye drops containing a disinfectant and antiinflammatory agents were regularly administered. Antiinflammatory agents, a painkiller, Insulin-like Growth Factor (IGF)-1, and other agents to stimulate wound healing were also given orally, and the patient was followed up every postoperative day for 1 week, then at 10 days and 20 days.

**Figure 1.**
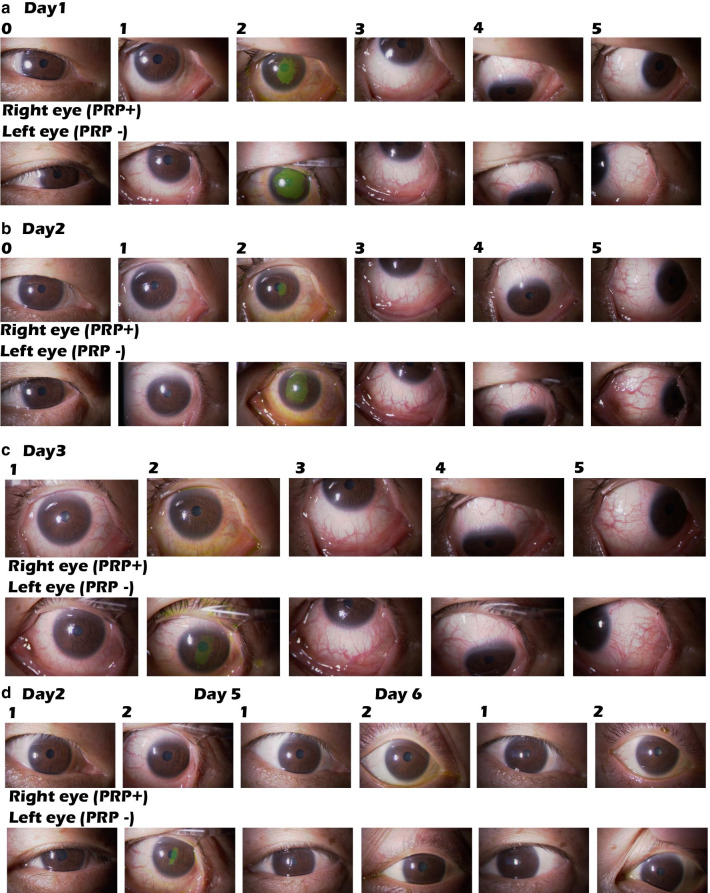
Photographic images of PRP-treated and untreated eyes after PRK. Images of the eyes showing all four angles (lane 1; front, lane 3; lower, lane 4; upper, lane 5; exterior) with eyelid speculum or without (lane 0) after operation. Corneal damage was visualized by staining with sodium fluorescein (lane 2). The PRP-treated right eye showed advanced wound healing compared to the untreated left eye.

To determine if an autologous platelet tissue graft may be beneficial for the promotion of wound healing after PRK, we used Gravitational Platelet Separation System with disposable separation tubes (GPSIII: BioMet Biologics) for preparation of PRP [10]. The PRP derived from the patient was administered to the right eye as eye drops every 2 or 3 hours for 1 week, then every 2–3 hours during the daytime for an additional week. The left eye was administered the same combination of eye drops excluding the PRP for one week after surgery. The patient felt pain immediately after PRK in both eyes, but within 2 hours pain was only felt in the untreated left eye up to two postoperative days. Consistently, palpebral fissure height was reduced in both eyes after PRK; however, recovery was seen after 4 days in the PRP-treated right eye, which was 3 days earlier than in the left eye (Fig.1). Pericorneal injection on the bulbar conjunctiva with superficial keratitis (SPK) was seen in both eyes at postoperative day 1, but was much less in the treated right eye compared to the untreated left eye from all four angles (front, lower, upper, exterior; Fig. 1).

Sodium fluorescein was used to evaluate corneal abrasion [11] in both eyes at postoperative day 1 (Fig. 1). The stained region in the treated right eye overlapped with the pupil area, whereas the abrasion in the left eye covered a much larger area (Fig. 1). At day 2, the stained area in the treated right eye was drastically diminished and became undetectable by day 3, while the left eye showed staining of several SPK spots until day 5. The presence of pericorneal injection followed a similar pattern as the corneal staining by sodium fluorescein.

**Figure 2.**
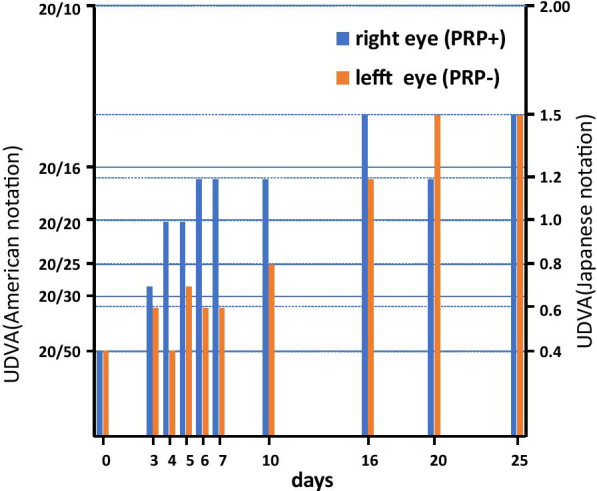
Visual outcomes after PRK in PRP-treated and untreated eyes. Uncorrected distance visual acuity (UDVA) was postoperatively monitored up to at 25 days, and the American and Japanese UDVA values are shown.

Myopic correction occurred as expected. Uncorrected distance visual acuity (UDVA) was postoperatively monitored starting at day 3 for 25 days (Fig.2). After 25 days, UDVA improved to 20/13 (1.5) in both eyes, but a difference between the PRK-treated versus untreated eye was evident in the time required for correction (Fig. 2). For the treated right eye, the UDVA improved to 20/17 (1.2) within one week, while the left eye required 20 days. The expected UDVA (20/13; 1.5) was achieved in the treated right eye at day 16, and for the left eye at day 20. Thus, all indications suggest that PRP treatment was beneficial to the patient by promoting recovery and corneal wound healing after PRK.

## Discussion

PRK and LASIK are widely accepted and performed as corneal refractive surgeries to improve vision. Owing to the differences in surgical procedures, both approaches have merits and demerits [1–5]. PRK does not require the creation of a flap, but takes more time for wound healing and imposes potential pain on the patient. Therefore, the enhanced promotion of wound healing would be expected to greatly benefit these patients.

Here, we used the GPS III system [10] to obtain PRP from the patient, as PRP has been clinically shown to promote wound healing in many postoperative settings [6–8]. To accurately assess the effects of PRP on wound healing, the untreated left eye was used as a control under informed consent. By monitoring perceived pain and overt corneal states with sodium fluorescein staining (Fig. 1), wound healing appeared to be enhanced by PRP treatment. Consistently, substantially better UDVA (20/17; 1.2) was achieved in the PRP-treated right eye within 1 week compared to the preoperative UDVA (20/50; 0.4 Fig. 2), while the untreated left eye required 20 days to achieve similar improvement. Thus, all indications assessed (Fig. 1) suggest that PRP treatment is potent for promoting corneal wound healing associated with PRK.

The corneal epithelium is a self-renewing tissue containing ample stem cells residing in the corneoscleral junction and limbus [1, 3]. Following corneal damage that disrupts the layered structure of the corneal epithelium and barrier function, epithelial regeneration is activated to preserve corneal transparency and vision. This is mediated through cell migration, proliferation, adhesion, and differentiation of stem cells. Such epithelial regeneration is achieved by a complicated and highly regulated interaction between growth factors/cytokines as well as extracellular matrix-induced signaling pathways [3]. Growth factors/cytokines such as PDGF, TGF-β, and EGF have been extensively studied to elucidate their roles in corneal epithelial regeneration [3, 12, 13]. As PRP is rich in such growth factors with clinical merit to avoid immune incompatibility by preparing from autologous blood of the patients [6], it is rational to suggest that PRP is advantageous in wound healing process after PRK. Indeed, there are a number of reports of the successful application of PRP in wound healing and tissue regeneration under pathological and physiological settings [6]. Furthermore, promotion of corneal nerve regeneration after PRK was experimentally observed in rabbit [14]. Thus, the present study is consistent with past findings in wound healing [1–3, 6]. However, it remains elusive why pain was relieved shortly after PRP treatment in the tested patient. Unidentified substance(s) in the PRP might be effective to abort pain.

While there is little information on the effects of PRP for epithelial regeneration after corneal refractive surgery, it can be hypothesized that PRP is beneficial, as it contains significant amounts of PDGF, TGF-β, and EGF [15]. In this respect, the present study can be viewed as an initial proof of concept for the therapeutic benefits of PRP in the cornea, as the first case in patients.

This study has a few limitations that should be noted. Since we presented results from only one patient, a greater number of patients should be treated to fully evaluate the effects of PRP on the wound healing process. Moreover, it is possible that other preoperative heath conditions might affect the outcomes of PRP treatment.

## Conclusions

PRP treatment was effective for promoting corneal wound healing after PRK, and PRP may be beneficial for patients undergoing corneal refractive surgeries.

## Data Availability

The datasets used and/or analyzed in this study are available upon reasonable request to the corresponding authors.

## Abbreviations

LASIK: Laser *in situ* keratomileusis
PRK: Photorefractive keratectomy
PRP: Platelet-rich plasma
SPK: Superficial keratitis
UDVA: Uncorrected distance visual acuity
GPS: Gravitational platelet separation
